# Supplementary figures and images for: Functional independence of endogenous μ- and δ-opioid receptors co-expressed in cholinergic interneurons
Source: eLife. 2021 Sep 3;10:e69740. doi: 10.7554/eLife.69740 (PMC8718112; doi:10.7554/eLife.69740)

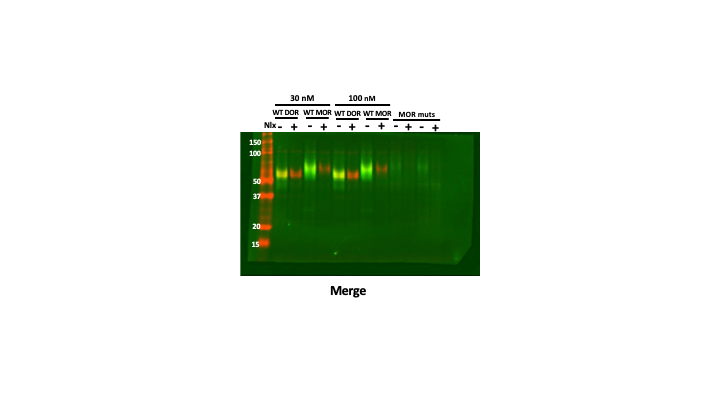

Supplement: Figure 1—source data 1. [file elife-69740-fig1-data1.zip › Figure 1_source data 1.tiff]

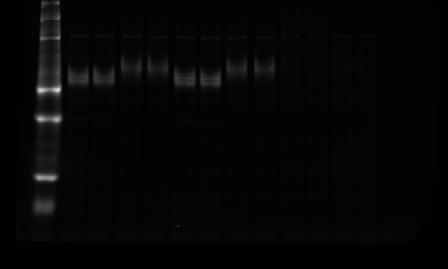

Supplement: Figure 1—source data 1. [file elife-69740-fig1-data1.zip › Fig_1C_MOR_DORmerge_source.tif]

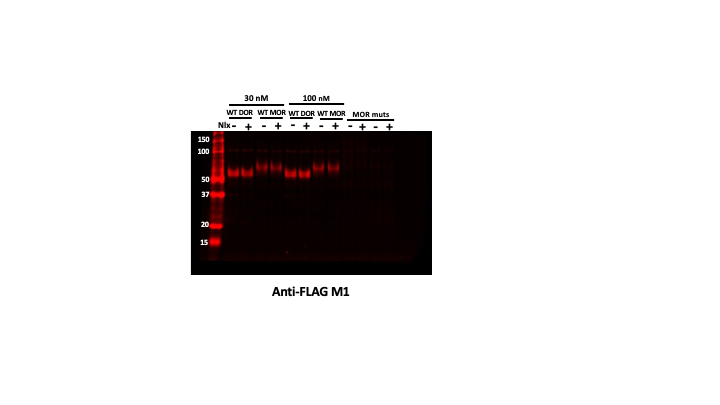

Supplement: Figure 1—figure supplement 1—source data 1. [file elife-69740-fig1-figsupp1-data1.zip › Figure 1_supplement 1_source data 1.tiff]

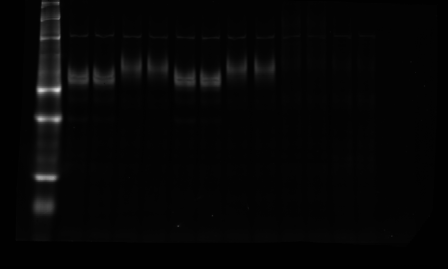

Supplement: Figure 1—figure supplement 1—source data 1. [file elife-69740-fig1-figsupp1-data1.zip › Supp_Fig1A_MOR_DOR_C1_source.tif]

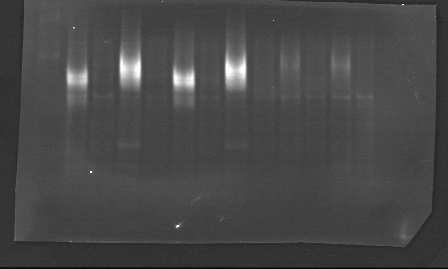

Supplement: Figure 1—figure supplement 1—source data 2. [file elife-69740-fig1-figsupp1-data2.zip › Supp_Fig1A_MOR_DOR_C2_source.tif]

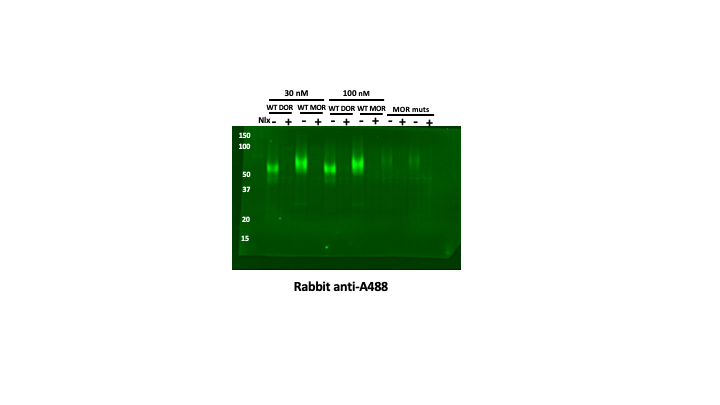

Supplement: Figure 1—figure supplement 1—source data 2. [file elife-69740-fig1-figsupp1-data2.zip › Figure 1_supplement 1_ source data 2.tiff]

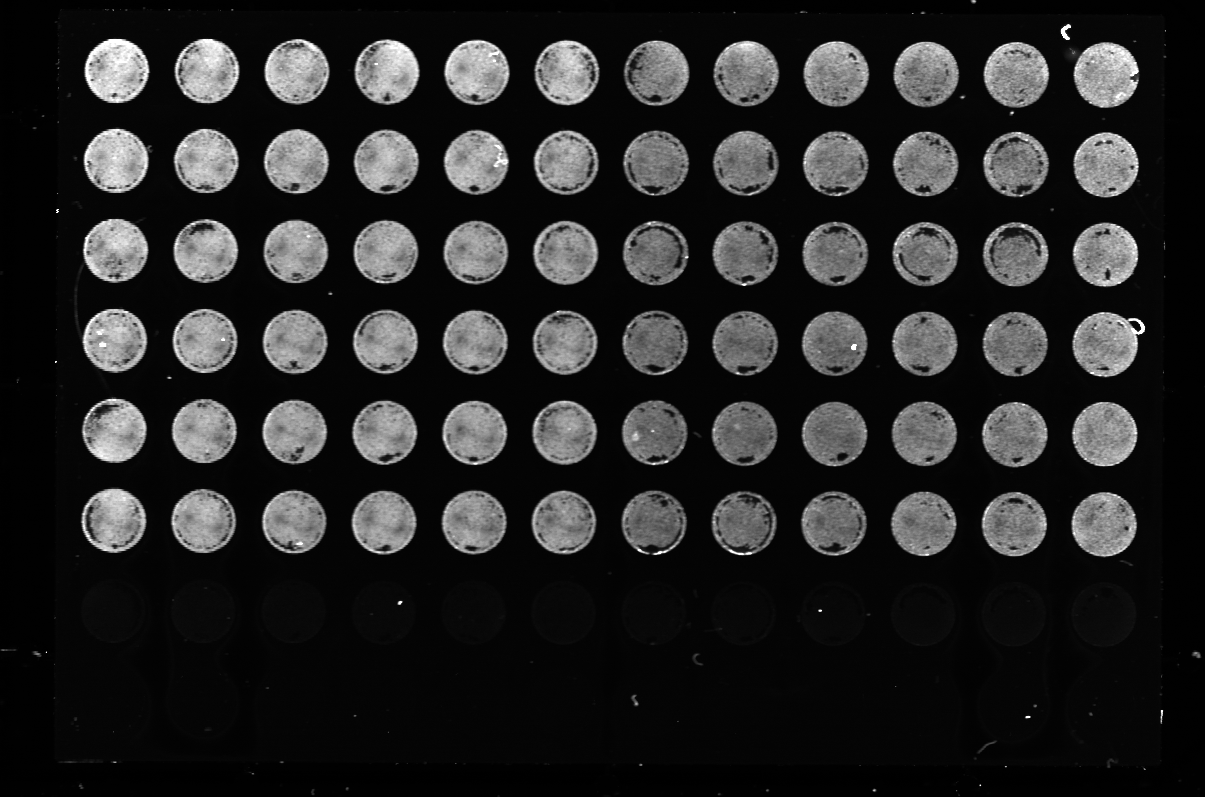

Supplement: Figure 1—figure supplement 1—source data 3. [file elife-69740-fig1-figsupp1-data3.zip › Supp_Fig1B_C1_source.tif]

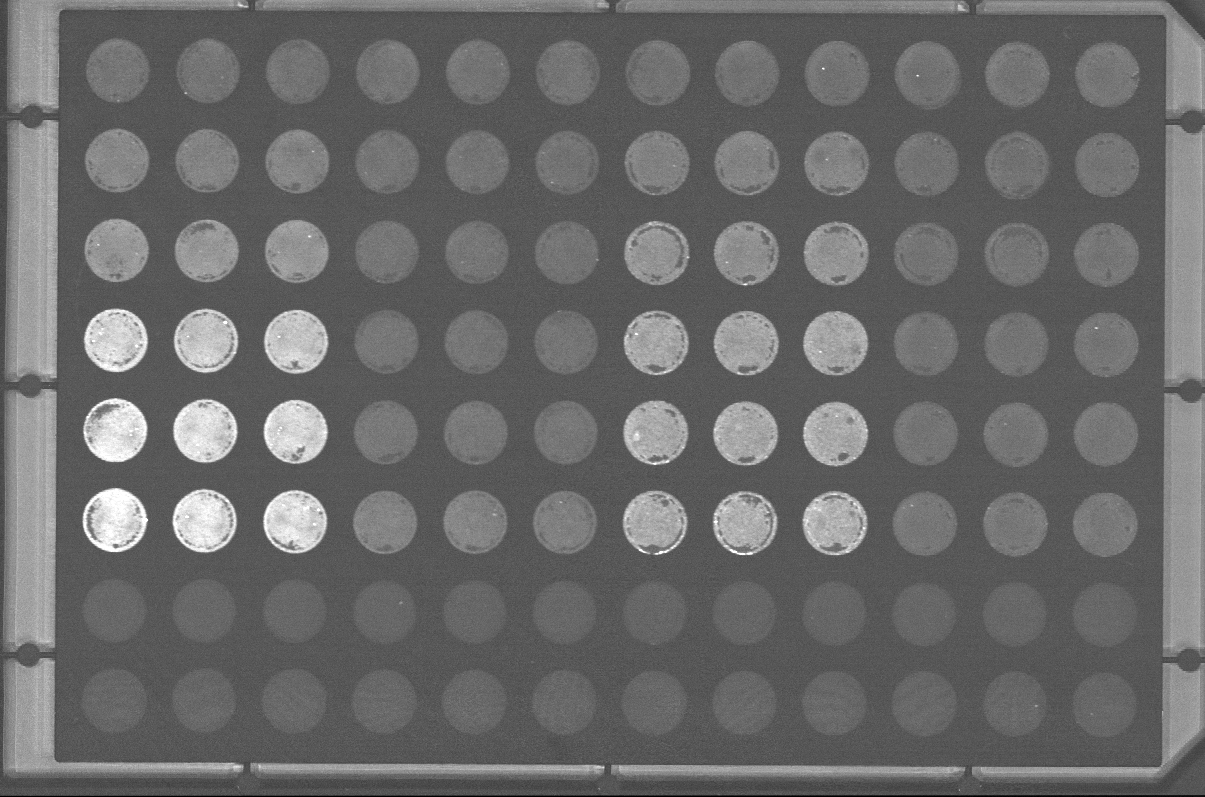

Supplement: Figure 1—figure supplement 1—source data 4. [file elife-69740-fig1-figsupp1-data4.zip › Supp_Fig1B_C2_source.tif]

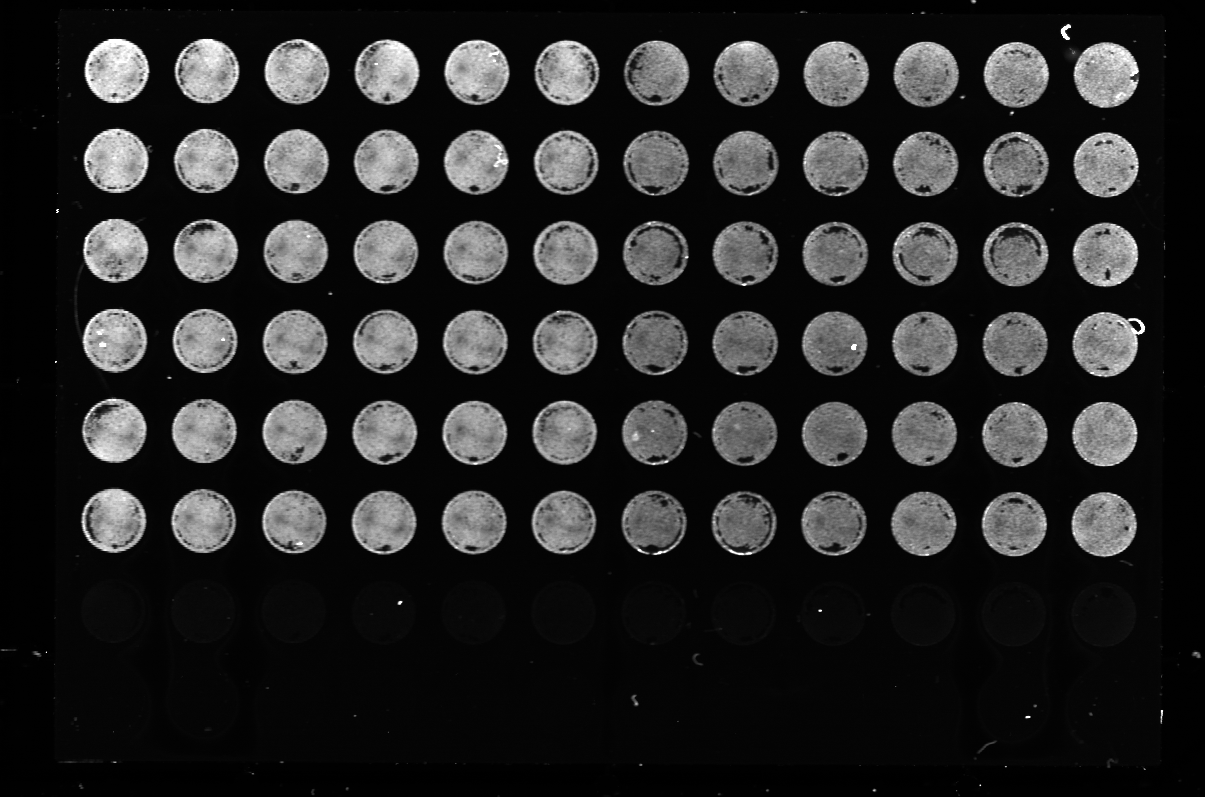

Supplement: Figure 1—figure supplement 1—source data 5. [file elife-69740-fig1-figsupp1-data5.zip › Supp_Fig1B_merge_source.tif]
